# Outcomes in COVID-19 Patients with Pneumonia Treated with High-Flow Oxygen Therapy and Baricitinib—Retrospective Single-Center Study

**DOI:** 10.3390/life13030755

**Published:** 2023-03-10

**Authors:** Dušanka Obradović, Milica Popović, Maja Banjac, Jelena Bulajić, Vladimir Đurović, Ivana Urošević, Aleksandra Milovančev

**Affiliations:** 1 Faculty of Medicine Novi Sad, University of Novi Sad, 21000 Novi Sad, Serbia; 2 Institute for Pulmonary Diseases of Vojvodina, 21204 Sremska Kamenica, Serbia; 3 Clinic of Nephrology and Clinical Immunology, University Clinical Centre of Vojvodina, 21000 Novi Sad, Serbia; 4 Urgent Care Center, University Clinical Centre of Vojvodina, 21000 Novi Sad, Serbia; 5 Clinic of Hematology, University Clinical Centre of Vojvodina, 21000 Novi Sad, Serbia; 6 Institute for Cardiovascular Diseases of Vojvodina, 21204 Sremska Kamenica, Serbia

**Keywords:** COVID-19, baricitinib, respiratory insufficiency, intensive care units, mortality

## Abstract

Background. The aim of the study was to assess the effect of baricitinib on 28-day all-cause mortality and the progression of respiratory failure in patients needing transfer to the intensive care unit (ICU) with COVID-19 pneumonia treated with high-flow oxygen therapy. Methods. This retrospective study included hospitalized patients with COVID-19 pneumonia treated with high-flow oxygen non-invasive ventilation receiving standard of care (SOC) or SOC in addition to baricitinib. Data on patients’ characteristics, pro-inflammatory markers, D dimer, and National Early Warning Score 2 (NEWS2) values were collected and compared between groups. The primary endpoint was 28-day all-cause in-hospital mortality and the secondary outcome was transfer to the ICU. Results. The study included 125 patients. The primary outcome was observed in 44.8% of them: 27% in the baricitinib group vs. 62% in the SOC group, *p* < 0.001. Transfer to the ICU ward was significantly lower in the baricitinib group: 29% vs. 81%, *p* < 0.001. A significant improvement was observed when the baricitinib group was compared to SOC in procalcitonin, CRP, D-dimer, neutrophil-to-lymphocyte ratio values, and NEWS2. Conclusion. Treatment with baricitinib in addition to SOC was associated with reduced mortality and a lower prevalence of transfer to the ICU in hospitalized patients with COVID-19 pneumonia treated with high-flow oxygen non-invasive therapy.

## 1. Introduction

The COVID-19 infection has caused the greatest “earthquakes” in the field of medicine in recent decades, both from the pandemic point of view as well as from the great ignorance related to the pathophysiology and therapy of this viral infection. From the beginning, reported mortality was high, especially for severely ill patients. In a systematic review of 27 cohort studies, in-hospital mortality was 32% in critically ill patients with COVID-19 for the year 2020 [1]. During the pandemic, therapeutic protocols changed, and in addition to the standard of care therapy (depending on the severity of the clinical presentation and respiratory failure), antiviral and anti-inflammatory drugs were introduced. Up-and-coming evidence showed that the alteration of the inflammatory response plays a crucial role in disease severity and mitigating a dysregulated immune system could prevent deterioration [2]. Among these emerging therapies, baricitinib has gained attention as a potential treatment due to its ability to inhibit Janus-associated kinase (JAK)1 and JAK2, as well as human Numb-associated kinases responsible for SARS-CoV-2 viral propagation, due to its anti-inflammatory, antiviral, and suppressing effects on the SARS-CoV-2 endocytosis [3]. This has been supported by the results of the two double-blind, randomized, placebo-controlled trials, which investigated the use of baricitinib either in combination with remdesivir (ACCT-2 study) or as a standalone therapy (COV-BARRIER study). The ACCT-2 trial reported that the baricitinib–remdesivir combination was superior to remdesivir alone in reducing recovery time and accelerating improvement in clinical status. In the COV-BARRIER study, baricitinib was associated with reduced mortality in hospitalized adults with COVID-19 [4,5]. As a result of these findings, the National Institutes of Health (NIH) Treatment Guidelines Panel has recommended the use of baricitinib in patients with rapidly increasing needs for higher respiratory support [6]. The use of oxygen therapy, including high-flow oxygen therapy (HFOT), has become a critical component of the standard of care for patients with severe COVID-19, as it can help to prevent the need for invasive mechanical ventilation and reduce the risk of mortality (over 75%) [7,8]. In November 2021, baricitinib became available for the treatment of hospitalized patients with COVID-19 infection in Serbia. The studies assessing baricitinib in severely ill patients are lacking. Providing additional data on baricitinib efficacy in this specific population could improve outcomes. The aim of the study was to assess the effect of baricitinib on 28-day all-cause mortality and the progression of respiratory failure in patients needing transfer to the intensive care unit (ICU) with COVID-19 pneumonia treated with HFOT. 

## 2. Materials and Methods

### 2.1. Study Design

The study protocol was approved by the Ethics Committee of the University Clinical Centre. The data were extracted from electronic medical records. The retrospective study included 125 patients hospitalized with laboratory-confirmed SARS-CoV-2 infection at the University Hospital in a period from November 2021 to April 2022. The inclusion criteria were as follows: ≥18 years old, with severe illness (SpO_2_ < 94% on room air at sea level, a ratio of arterial partial pressure of oxygen to fraction of inspired oxygen (PaO_2_/FiO_2_) < 300 mmHg, respiratory rate > 30 breaths/min, or lung infiltrates >50%) [6], and treated with HFOT (NIAID OS 6) [9]. The patients were divided into 2 groups: a group treated with baricitinib plus standard of care (SOC) and a group treated with SOC alone. The first group received 4 mg of baricitinb orally once daily for 14 days (patients with abnormal laboratory values where modification of the baricitinib dose or interruption of the baricitinib were indicated were excluded from the study). SOC included systemic corticosteroids (dexamethasone 6 mg once daily via i.v.) and the prevention of venous thromboembolism with low-molecular-weight heparin (LMWH) and gastric ulcer prophylaxis. Demographic characteristics and comorbidities, values of proinflammatory markers, D dimer, neutrophil-to-lymphocyte ratio (NLR), National Early Warning Score 2 (NEWS2) values on Days 1 (the day when the baricitinib was introduced in therapy), 4, 7, and 10, and vaccination status were investigated. Primary end-point was 28-day all-cause in-hospital mortality, and the second outcome was transfer to the intensive care unit (ICU) with a need for non-invasive or invasive mechanical ventilation. All patients were tracked for serious adverse events (SAEs) and no SAEs were present. The study was approved by the Ethical Committee of the University Clinical Centre of Vojvodina, Novi Sad (protocol code: 00-40, date of approval: 9 February 2023).

### 2.2. Statistical Analysis

The normality of the data was assessed using the Kolmogorov–Smirnov test. Non-normally distributed continuous variables were presented as the median (Mdn) with the first and the third quartile (Q1–Q3), while categorical variables were presented as relative numbers (frequencies). Differences between groups were assessed using the Wilcoxon rank sum test for continuous data and the chi-square test or the Fischer exact test for categorical data. Statistical significance was set at *p*-value < 0.05. Discriminatory capacity, e.g., predictive strength of some laboratory predictors for primary and secondary outcomes, was evaluated via the area under the receiver operating characteristic (ROC) curve (AUC) using the bootstrapping method. The positive predictive value (PPV) and negative predictive value (NPV) were calculated for each predictor, based on sensitivity, specificity, and disease prevalence. Youden’s index was used for cut-off selection. No imputations were used for the missing data. Statistical analysis was performed using the RStudio 2022.07.1 + 554 “Spotted Wakerobin” Release.

## 3. Results

### 3.1. Baseline Patients’ Characteristics

Baseline patients’ characteristics are presented in Table 1. When baricitinib and SOC groups were compared there were no gender (52% vs. 68% males, *p* = 0.06) or age differences (66.5 (55–74) vs. 68 (60–71), *p* = 0.93). The most common comorbidity was hypertension in both groups and more than 30% of patients in both groups were obese. There were no significant differences in the prevalence of comorbidities between groups and no significant differences regarding the vaccinal status. No serious adverse events (SAEs) were registered in patients treated with baricitinib. 

### 3.2. Laboratory Findings

Inflammatory parameters C reactive protein (CRP) and Procalcitonin (PCT), as well as D-dimer values, were significantly decreased in the measuring points in the baricitinib group (Table 2). CRP levels were significantly lower on Day 4, with a median of 42.2 (24.0–78.3) vs. 78.8 (36.3–124.7), *p* = 0.002; Day 7, median 28.5 (24.0–78.3) vs. 80.2 (36.3–124.7), *p* < 0.001; and Day 10, median 12.1 (4.1–60.8) vs. 83.5 (32.1–210.8), *p* < 0.001. Procalcitonin levels showed a significant drop in the baricitinib group compared to the SOC group with the lowest values going as low as a median of 0.04 on Days 7 and 10 ((0.03–0.10), (0.02–0.06), respectively) compared to a median of 12 (0.08–0.48) on Day 7 and a median of 18 (0.08–1.37) on Day 10, *p* < 0.001. The levels of D-dimer were also significantly lower in the baricitinib group throughout the treatment period on Day 1, median 1.0 (0.6–1.3) vs. 1.6 (0.9–2.8), *p* = 0.002; Day 4, median 1.0 (0.8–2.2) vs. 2.5 (1.4–9.7), *p* < 0.001; Day 7, median 1.3 (0.8–2.9) vs. 2.4 (1.4–5.4), *p* = 0.001; and Day 10, median 1.0 (0.5–1.9) vs. 2.4 (1.1–5.3), *p* < 0.001 (Table 2).

The lymphocyte count was significantly higher in the baricitinib group on all days: on Day 1, the median was 0.8 (0.6–0.9) vs. 0.6 (0.5–0.9), *p* = 0.009; on Day 4, the median was 1.1 (0.7–1.5) vs. 0.7 (0.4–0.9), *p* < 0.001; on Day 7, the median was 1.0 (0.7–1.6) vs. 0.7 (0.4–1.0), *p* < 0.001; and on Day 10, the median was 1.2 (0.8–1.9) vs. 0.6 (0.4–0.9), *p* < 0.001. The neutrophil count was significantly lower in the baricitinib group on Day 4, with a median of 8.2 (5.8–10.2) vs. 9.8 (6.9–13.5), *p* = 0.026, with a significant difference on Day 7, and the highest difference on Day 10, median 7.9 (6.1–11.9) vs. 12.6 (8.6–16.7), *p* < 0.001. The neutrophil-to-lymphocyte ratio (NLR) was significantly lower in the baricitinib group, while the highest drop was observed on Day 10, with a median of 6.0 (4.0–11.0) vs. 20.0 (12.0–9.0), *p* < 0.001, as shown in Table 3. 

NEWS2 showed a significant drop from Day 1 up to Day 10, compared to the SOC group, with the lowest value on Day 10, with a median of 2.0 (2.0–3.0) vs. 3.5 (2.0–5.0), *p* = 0.003 (Table 4). 

### 3.3. Outcomes

The primary outcome (28-day all-cause in-hospital mortality) was observed in 56 patients (44.8%): 17 (27%) in the baricitinib group vs. 39 (62%) in the SOC group, *p* < 0.001. 

The prevalence of the ICU ward transfer was also significantly lower in the baricitinib group: 29% compared to 81% of patients in the SOC group (*p* < 0.001) (Table 5). There were no SAEs reported. 

#### 3.3.1. Predictive Roles of the NLR and NEWS2 for the Primary Outcome

**Figure 1:**
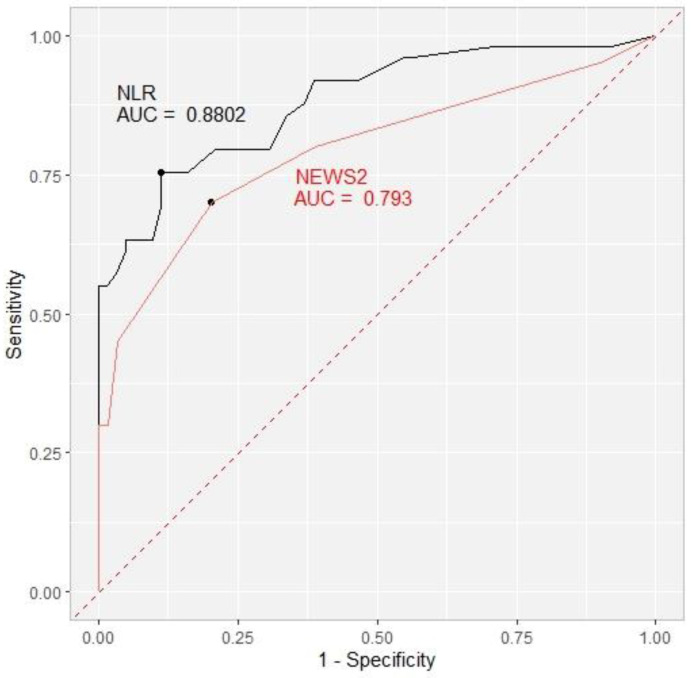
Receiver operating characteristic curve (ROC) showing the ability of the NLR (black) and NEWS2 (red) to predict the primary outcome on Day 10 with the area under the curve (AUC) included. NLR = neutrophil-to-lymphocyte ratio. NEWS2 = National Early Warning Score 2.

We decided to investigate the prognostic role of the NLR as a simple marker of baricitinib’s anti-inflammatory effect and NEWS 2 as an indicator of clinical improvement in relation to outcomes. On Day 1 and Day 4, the NLR predictive cut-off value was 10 with an area under the curve (AUC) of 0.615 and 0.776, respectively. On Day 7, the NLR cut-off value rose to 13 with a higher AUC of 0.849 and, finally, on Day 10, the NLR cut-off of 17 exhibited the highest AUC of 0.880. The cut-off values for NEWS2 were 4 on Day 1, Day 4, and Day 10 with an AUC of 0.604, 0.665, and 0.793, respectively, and 3 on Day 7 (AUC 0.752) (Table 6, Figure 1). 

#### 3.3.2. Predictive Roles of the NLR and NEWS2 for the Secondary Outcome

**Figure 2:**
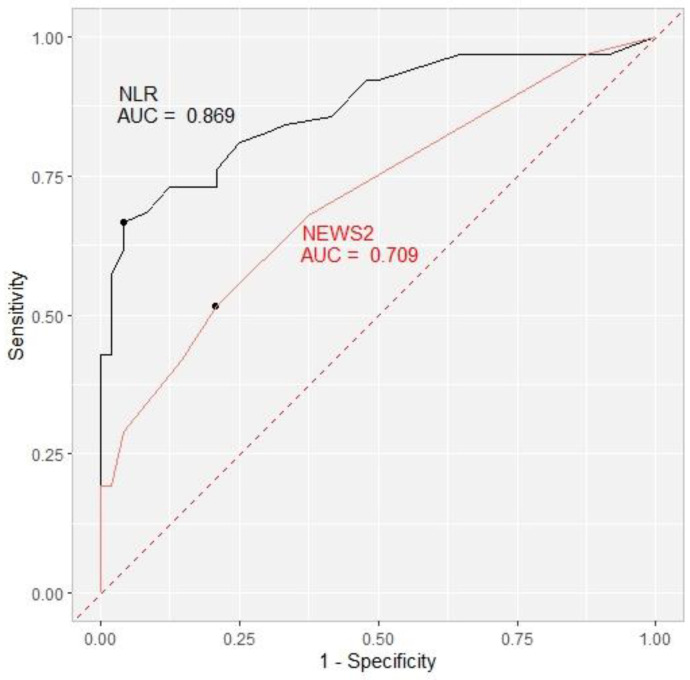
Receiver operating characteristic curve (ROC) showing the ability of the NLR (black) and NEWS2 (red) to predict the secondary outcome on Day 10 with area under the curve (AUC) included. NLR = neutrophil-to-lymphocyte ratio. NEWS2 = National Early Warning Score.

On Day 1, the cut-off value for the NLR was 10 with an area under the curve of 0.658. Over the course of treatment, the NLR cut-off values rose to 12 on Day 4, with an AUC of 0.775, 14 with an AUC of 0.819 on Day 7, and finally 17 with the highest AUC of 0.869 on the 10th day. The cut-off value for NEWS2 for the prediction of the secondary outcome was 4 on Day 1 and Day 4, with an AUC of 0.567 and 0.594, respectively. On Days 7 and 10, the cut-off value decreased to 3 (AUC 0.650 and 0.709, respectively) (Table 7, Figure 2). 

## 4. Discussion

In this study, the number of patients with respiratory failure progression (a need for non-invasive or invasive mechanical ventilation with transfer to the ICU) was significantly lower in the baricitinib group in contrast to the SOC group (29% vs. 81%, respectively). The 28-day all-cause mortality rate was also significantly reduced in patients treated with baricitinib compared to the SOC group (27% vs. 62%, respectively, *p* < 0.001). The results of our study are similar to the results of the COV-BARRIER study [5], where the use of baricitinib plus SOC showed a 38.2% relative reduction in mortality compared to SOC (dexamethasone) (hazard ratio (HR) of 0.57, (95% CI: 0.41–0.78), nominal *p* = 0.0018) in the group of patients who required oxygen therapy (NIAID OS 5). The positive results from this study were also present in the group that required HFOT or non-invasive ventilation (NIAID OS 6), with a difference in the 28-day all-cause mortality between the baricitinib and placebo groups (HR of 0.52 (95% CI: 0.33–0.80; *p* = 0.0065)). In the COV-BARRIER study, the efficacy of baricitinib regarding the progression to higher respiratory support (HFOT, NIV, and invasive mechanical ventilation) as a primary outcome was not proven. In our study, transfer to the ICU was significantly lower in the baricitinib group. The difference between the results could be explained by the enrollment criteria regarding the level of respiratory support at baseline as our group of patients was uniform in relation to the level of needed respiratory support. 

In the exploratory, randomized, placebo-controlled study on the COV-BARRIER study group, patients treated with invasive mechanical ventilation or extracorporeal membrane oxygenation and baricitinib [9] had a significantly lower rate of death (20 of 51 patients died (38%)) compared to the placebo group (29 of 50 patients died (58%)). In the RECOVERY trial [10], the majority of included patients were treated with simple oxygen therapy (67% in the baricitinib group and 68% in the SOC group), while non-invasive ventilation was less frequent (24% vs. 23%, respectively). In addition to baricitinib, patients were treated with corticosteroids (96% vs. 85%, respectively), remdesivir, and tocilizumab (more than 20% of the patients in both groups). The primary outcome was 28-day mortality and a significant reduction in the mortality rate was present in the baricitinib (12%) vs. the SOC group (14%), (age-adjusted rate ratio (RR) 0.87, (95% CI: 0.77–0.99); *p* = 0.028). The progression to invasive mechanical ventilation was also significantly lower in patients treated with baricitinib (16%) vs. the SOC group (17%), (RR 0.89, (95% CI: (0.81–0.98)), *p* = 0.016). The RECOVERY trial was included in the meta-analysis alongside the previous eight randomized controlled trials (with 43% mortality reduction) of the JAK inhibitor [10]. Adding the RECOVERY trial to these trials resulted in a reduction in 28-day mortality by one fifth. 

After the results from the artificial intelligence repository [11] and studies about the anti-inflammatory effects of JAK inhibitors [12,13], baricitinib was proposed as a potential drug of choice in the treatment of COVID-19 [14]. Proinflammatory markers, besides diagnostic significance, also have prognostic importance in patients with COVID-19. In the study by colleagues from China [15], the CRP, PCT, and NLR were investigated in relation to their diagnostic and prognostic significance for COVID-19 mortality in groups related to the severity of the disease. The levels of PCT and the NLR were higher in the severe and critically ill patients and the levels of PCT, CRP, and the NLR had prognostic significance for COVID-19 mortality. Furthermore, in this study, levels of CRP (≥52.14 mg/L) and PCT (≥0.10 ng/mL) were independent risk factors for mortality (after adjusting covariates) with HRs of 52.68 (95% CI: 1.77–1571.66) and 5.47 (95% CI: 1.04–28.72), respectively, while the NLR values showed no statistical significance in relation to mortality. The NLR was investigated in systematic reviews and meta-analyses as being an indicator of COVID-19 disease severity, as well as for its diagnostic and prognostic roles [16,17]. These studies found that the NLR levels were significantly higher in patients with COVID-19 infection, particularly in those with moderate to severe disease, and that patients who died had significantly higher NLR levels.

The inflammatory parameters CRP and PCT showed significantly lower values in the baricitinib group at various timepoints. Specifically, in a retrospective longitudinal multicenter study conducted in Italy on patients with moderate pneumonia due to COVID-19 and treated with baricitinib and lopinavir-ritonavir, CRP levels were significantly reduced in the first week (*p* = 0.003) as well as in the second week (*p* = 0.001) compared to patients treated with standard therapy (antiviral agents plus hydroxychloroquine) [18]. However, in this study, PCT levels did not show significant improvement in the baricitinib group after the first or second week. In contrast, our study found significantly lower PCT levels in the baricitinib group.

In our study, the levels of D-dimer were also significantly lower in the baricitinib group throughout the treatment period. Regarding the baricitinib effects on the D-dimer and CRP values, the results of our study are very similar to those of the retrospective study by Thoms et al. [19] which included 45 patients with COVID-19 pneumonia. We report significantly lower levels of CRP and D-dimer in the baricitinib group over the course of treatment which are comparable to their results. A retrospective, observational study on 31 patients performed in India [20] studied the effects of remdesivir and baricitinib plus corticosteroids combination therapy on oxygen requirements, clinical outcomes, and the values of CRP, interleukin 6, NLR, ferritin, and D-dimer. The oxygen requirements were significantly lower during the treatment regardless of age and comorbidities. The CRP levels showed a significant reduction on Days 1, 3, 5, and 7 (83.9 to 32.3 mg/L with *p* value of 0.0001 at 95% confidence interval) with similar results in relation to the outcomes (79.8 to 26.45 with *p* value of 0.002 in survivors’ group). D-dimer levels in this study exhibited no significant decrease.

The NLR has been used as a marker of inflammation with the ability of having a prognostic role regarding morbidity and mortality in many conditions [21,22,23]. It was proposed as a screen tool for high-risk patients in the COVID-19 pandemic [24,25] because of its simplicity and low cost. In a systematic review and meta-analysis [26] where the primary outcome was to determine the diagnostic and prognostic accuracy of the NLR in COVID-19 patients, the authors concluded that the NLR was significantly higher in SARS CoV-2 positive patients and has shown a good predictive ability in relation to mortality (sensitivity 83%, specificity 80%). In a review of screening, diagnostic, and prognostic tests for COVID-19, Ulinici et al. [27] emphasized the diagnostic and prognostic significance of several typical hematologic findings, including the neutrophil-to-lymphocyte ratio (NLR). Patients with COVID-19 were found to have higher levels of the NLR compared to non-COVID-19 patients, and non-survivors were found to have higher levels of the NLR compared to survivors. In our study, we investigated the changes in the NLR during the treatment with baricitinib and this marker was significantly lower in the baricitinib group, while the highest drop was observed on Day 10. Regarding the ability of the NLR to predict outcomes, on Day 1 the cut-off value was 10 for differentiating both mortality and transfer to the ICU. After the period of 10 days, the cut-off value rose to 17 for predicting both primary and secondary outcomes which can be explained by the fact that the median value of the NLR rose significantly in the SOC group, from 12.0 to 20.0, while the NLR in the baricitinib group exhibited a significant drop, from 10.0 to 6.0 on Day 10, and by the fact that the overall mortality was significantly higher in the SOC group altogether.

NEWS2 [28] is one of the Early Warning Scores proposed for the early identification of deteriorating COVID-19 patients. According to the results of a retrospective analysis [29] of 296 hospitalized patients with PCR-confirmed COVID-19 infection, NEWS2 ≥ 5 had a sensitivity of 98% and specificity of 28% for predicting the first serious deterioration, with a higher negative predictive value (NPV) of 96%, compared to the Modified Early Warning Score (MEWS) and quick Sepsis-Related Organ Failure Assessment (qSOFA). We used NEWS2 for clinical monitoring of the patients with COVID-19. In the secondary analysis of the Adaptive COVID-19 Treatment Randomized Trial-2 (ACCT-2) [30], the authors revisited the effects of baricitinib plus remdesivir on the clinical improvement in the patients that required oxygen therapy at baseline. Patients in the OS6 group (treated with HFOT and NIV) who received baricitinib had the greatest benefit in terms of clinical improvement speed and prevention of clinical deterioration, resulting in fewer transfers to the ICU as measured using the ordinal scale (OS). In our study, NEWS2 was proven to be a significant predictor of both primary and secondary outcomes. This is comparable to several other studies that assessed NEWS2 predictive strength in COVID-19 patients [31,32,33,34]. In a multicenter study of 1288 COVID-19 patients treated in hospitals in the west of England [31], high NEWS2 values upon admission and the highest measured levels of NEWS2 during the in-hospital treatment were predictive for deterioration and ICU transfer, length of stay, and mortality. The study included all COVID-19 positive patients admitted to the hospitals regardless of the severity of the disease and with no specified treatment regimen. The highest observed AUC for predicting the mortality was on the second day of the hospitalization (0.77, (95% CI: 0.70–0.84)). In our study, the AUC for NEWS2 ranged from 0.604 up to 0.793 for the primary outcome prediction, implying that this score became a better predictor for mortality as the treatment progressed. The explanation for this lies in the fact that at the baseline, our research included the patients that were classified as being severely ill (OSC 6), and over the course of the treatment, the clinical status of the treated patients improved noticeably, in contrast to the SOC group, which also had a higher marked mortality. Similar findings were observed for the secondary outcome, where the AUC ranged from 0.567 to 0.709. In another multicenter study [33], the evaluation of confusion, urea, respiratory rate, blood pressure, age above or below 65 years (CURB-65), and NEWS2 and Quick Sequential (Sepsis-Related) Organ Failure Assessment (qSOFA) scores was performed in COVID-19 patients. ICU admission, early in-hospital mortality (within 72 h), and all-cause mortality prediction ability were tested and NEWS2 was found to be superior in predicting early death with 92% sensitivity and a high NPV. In the retrospective study of patients admitted to the referral center in Italy [35], the authors tested the accuracy of several early warning systems to predict clinical outcomes. NEWS2 did not show an advantage for predicting ICU admission or death. The findings of our study, however, confirm the prognostic significance of NEWS2 in severely ill COVID-19 patients. These results highlight the potential utility of NEWS2 as a tool for monitoring disease progression and the response to treatment in this patient population.

COVID-19 is a disease with very high risk for thromboembolic complications [36,37,38], despite the anticoagulation prophylaxis. The prothrombotic effects of baricitinib, particularly in patients with rheumatoid arthritis (RA), have been extensively studied given its role as a JAK inhibitor [39]. The safety outcomes of baricitinib in rheumatology, dermatology, and patients with COVID-19 have been the subject of several clinical trials, as discussed in a recent review [40]. One of these trials, the COV-BARRIER study [3], involved randomizing patients 1:1 to receive either baricitinib plus standard of care (SOC) or placebo plus SOC (which included dexamethasone and antiviral drugs, mainly remdesivir). The study found that adverse events were present in more than 40% of patients in both groups, with the frequency of venous thromboembolisms (VTEs) being almost similar in the baricitinib and placebo groups (2.7% vs. 2.5%, respectively). However, there were slightly more patients with pulmonary embolisms in the baricitinib group (13 patients, 1.7%) than in the SOC group (9 patients, 1.2%). Serious infections were also present in both groups, with 64 (9%) in the baricitinib group and 74 (10%) in the SOC group, and opportunistic infections were observed in 1% of patients in both groups. The ACCT-2 trial [2] also examined the safety profile of baricitinib in combination with remdesivir. The results showed that both the combination group and the control group (remdesivir plus placebo) had a similar number of patients experiencing serious or non-serious adverse events of venous thromboembolism (21 patients, 4.1%, and 16 patients, 3.1%, respectively). However, there were fewer cases of new infections in the baricitinib group (30 patients, 5.9%) than in the control group (57 patients, 11.2%). Regarding serious adverse events (SAEs), our study did not report any cases of venous thromboembolism or cardiovascular events such as myocardial infarction or stroke in patients treated with baricitinib, either during the course of treatment or after discontinuation. Additionally, there were no instances of new infections recorded amongst our patients.

### Study Limitations

This study has several limitations. Although the primary and secondary outcomes were achieved, the study had a retrospective character and thus has all of the limitations of a retrospective study. In addition, it included a relatively small number of patients treated with baricitinib; so, these conclusions must be interpreted with caution for other subpopulations of patients with COVID-19. Finally, other factors such as age and comorbidity could have had an impact on outcomes. 

## 5. Conclusions

In conclusion, our study demonstrates that adding baricitinib to the standard of care in hospitalized patients with COVID-19 pneumonia receiving high-flow oxygen therapy is associated with a significant reduction in 28-day mortality and a lower prevalence of respiratory failure progression and transfer to the ICU. These findings highlight the potential benefits of baricitinib in preventing adverse outcomes in severely ill COVID-19 patients. Our study adds to the growing body of evidence supporting the use of baricitinib in the treatment of COVID-19 and provides valuable insights into its potential therapeutic benefits in this patient population. Further research is needed to confirm these findings and optimize the use of baricitinib in the management of COVID-19.

## Figures and Tables

**Table 1:** Characteristics of patients at baseline.

Parameter	Baricitinib, *n* = 62 ^2^	SOC ^3^, *n* = 63 ^2^	*p*-Value ^1^
Sex (male)	32 (52%)	43 (68%)	0.058
Age	66.5 (55–74)	68 (60–71)	0.927
BMI > 30 ^4^	24 (39%)	20 (32%)	0.415
Hypertension	45 (73%)	47 (75%)	0.798
Diabetes	18 (29%)	21 (33%)	0.604
COPD ^5^	9 (15%)	10 (16%)	0.833
Cardiovascular diseases	13 (21%)	14 (22%)	0.865
CKD ^6^	1 (1.6%)	4 (6.3%)	0.365
Oncologic diseases	4 (6.5%)	6 (9.5%)	0.744
Dyslipidemia	5 (8.1%)	6 (9.5%)	0.773
History of CVI ^7^	2 (3.2%)	2 (3.2%)	1
Vaccinated	14 (23%)	23 (37%)	0.088

^1^ Pearson’s Chi-squared test; Wilcoxon rank sum test; Fisher’s exact test, ^2^ *n* (%), Mdn (Q1–Q3) Mdn = Median, ^3^ SOC = Standard Of Care, ^4^ BMI = Body-Mass Index, ^5^ COPD = Chronic Obstructive Pulmonary Disease, ^6^ CKD = Chronic Kidney Disease, ^7^ CVI = Cerebrovascular Insult.

**Table 2:** Laboratory parameters measured on Days 1, 4, 7, and 10.

Laboratory Parameter	Baricitinib, *n* = 62 ^2^	SOC, *n* = 63 ^2^	*p*-Value ^1^
C-reactive protein—CRP (mg/L)			
CRP Day 1	104.8 (59.1–149.0)	108.4 (61.9–144.6)	0.824
CRP Day 4	42.2 (24.0–78.3)	78.8 (36.3–124.7)	0.002 *
CRP Day 7	28.5 (8.6–74.0)	80.2 (33.3–178.2)	<0.001 *
CRP Day 10	12.1 (4.1–60.8)	83.5 (32.1–210.8)	<0.001 *
Procalcitonin—PCT (ng/mL)			
PCT Day 1	0.09 (0.05–0.17)	0.12 (0.08–0.46)	0.003 *
PCT Day 4	0.05 (0.04–0.09)	0.11 (0.07–0.43)	<0.001 *
PCT Day 7	0.04 (0.03–0.10)	0.12 (0.08–0.48)	<0.001 *
PCT Day 10	0.04 (0.02–0.06)	0.18 (0.08–1.37)	<0.001 *
D-dimer—DD (ng/mL)			
DD Day 1	1.0 (0.6–1.3)	1.6 (0.9–2.8)	0.002 *
DD Day 4	1.0 (0.8–2.2)	2.5 (1.4–9.7)	<0.001 *
DD Day 7	1.3 (0.8–2.9)	2.4 (1.4–5.4)	0.001 *
DD Day 10	1.0 (0.5–1.9)	2.4 (1.1–5.3)	<0.001 *

^1^ Wilcoxon rank sum test; ^2^ Mdn (Q1–Q3). * show that the *p* value is <0.05.

**Table 3:** Lymphocyte, neutrophil count, and NLR values measured on Days 1, 4, 7, and 10.

Laboratory Parameter	Baricitinib, *n* = 62 ^2^	SOC, *n* = 63 ^2^	*p*-Value ^1^
Lymphocyte count—Ly (×10^9^)			
Ly Day 1	0.8 (0.6–0.9)	0.6 (0.5–0.9)	0.009 *
Ly Day 4	1.1 (0.7–1.5)	0.7 (0.4–0.9)	<0.001 *
Ly Day 7	1.0 (0.7–1.6)	0.7 (0.4–1.0)	<0.001 *
Ly Day 10	1.2 (0.8–1.9)	0.6 (0.4–0.9)	<0.001 *
Neutrophil count—Ne (×10^9^)			
Ne Day 1	7.6 (5.8–9.9)	8.6 (6.1–11.2)	0.326 *
Ne Day 4	8.2 (5.8–10.2)	9.8 (6.9–13.5)	0.026 *
Ne Day 7	9.0 (7.5–11.9)	12.0 (9.7–15.9)	0.002 *
Ne Day 10	7.9 (6.1–11.9)	12.6 (8.6–16.7)	<0.001 *
Neutrophil-to-lymphocyte ratio—NLR			
NLR Day 1	10.0 (6.0–13.0)	12.0 (8.0–21.0)	0.016 *
NLR Day 4	8.0 (5.0–13.0)	15.0 (9.0–22.0)	<0.001 *
NLR Day 7	8.0 (5.0–18.0)	19.0 (11.0–34.0)	<0.001 *
NLR Day 10	6.0 (4.0–11.0)	20.0 (12.0–39.0)	<0.001 *

^1^ Wilcoxon rank sum test; ^2^ Mdn (Q1–Q3). * show that the *p* value is <0.05.

**Table 4:** NEWS2 on Days 1, 4, 7, and 10.

National Early Warning Score—NEWS2	Baricitinib, *n* = 62 ^2^	SOC, *n* = 63 ^2^	*p*-Value ^1^
NEWS2 Day 1	4.00 (3.00–5.00)	4.00 (3.00–6.00)	0.190
NEWS2 Day 4	3.00 (2.00–4.25)	4.00 (3.00–6.00)	0.008 *
NEWS2 Day 7	3.00 (2.00–4.00)	3.50 (2.75–5.00)	0.047 *
NEWS2 Day 10	2.00 (2.00–3.00)	3.50 (2.00–5.00)	0.003 *

^1^ Wilcoxon rank sum test; ^2^ Mdn (Q1–Q3). * show that the *p* value is <0.05.

**Table 5:** Primary and secondary outcomes.

Outcomes	Baricitinib, *n* = 62 ^2^	SOC, *n* = 63 ^2^	*p*-Value ^1^
Primary outcome (death)	17 (27%)	39 (62%)	<0.001 *
Secondary outcome (ICU ward admission)	18 (29%)	51 (81%)	<0.001 *

^1^ Pearson’s chi-squared test; ^2^ *n* (%). * show that the *p* value is <0.05.

**Table 6:** Predictive role of the NLR and NEWS 2 for primary outcome via ROC curve analysis.

Predictor	Cut-Off	Sensitivity	Specificity	PPV ^1^	NPV ^2^	AUC ^3^ (95% CI ^4^)
Neutrophil-to-lymphocyte ratio—NLR						
NLR Day 1	10	0.69	0.52	0.54	0.67	0.615 (0.54–0.70)
NLR Day 4	10	0.82	0.62	0.64	0.81	0.776 (0.70–0.84)
NLR Day 7	13	0.82	0.72	0.70	0.83	0.849 (0.79–0.90)
NLR Day 10	17	0.75	0.88	0.83	0.81	0.880 (0.82–0.94)
National Early Warning Score—NEWS2						
NEWS Day 1	4	0.69	0.48	0.52	0.66	0.604 (0.53–0.69)
NEWS Day 4	4	0.65	0.66	0.57	0.73	0.665 (0.58–0.75)
NEWS Day 7	3	0.91	0.48	0.47	0.91	0.752 (0.67–0.83)
NEWS Day 10	4	0.70	0.80	0.54	0.89	0.793 (0.67–0.89)

^1^ PPV = positive predictive value, ^2^ NPV = negative predictive value, ^3^ AUC = area under the curve, ^4^ CI = confidence index.

**Table 7:** Predictive role of the NLR and NEWS for secondary outcome via ROC curve analysis.

Predictor	Cut-Off	Sensitivity	Specificity	PPV ^1^	NPV ^2^	AUC ^3^ (95% CI ^4^)
Neutrophil-to-lymphocyte ratio—NLR						
NLR Day 1	10	0.69	0.56	0.67	0.59	0.658 (0.57–0.53)
NLR Day 4	12	0.64	0.78	0.79	0.63	0.775 (0.71–0.84)
NLR Day 7	14	0.71	0.78	0.80	0.68	0.819 (0.76–0.88)
NLR Day 10	17	0.67	0.96	0.95	0.68	0.869 (0.81–0.92)
National Early Warning Score—NEWS2						
NEWS2 Day 1	4	0.67	0.48	0.61	0.54	0.567 (0.48–0.65)
NEWS2 Day 4	4	0.59	0.65	0.64	0.60	0.594 (0.51–0.69)
NEWS2 Day 7	3	0.77	0.45	0.53	0.71	0.650 (0.56–0.74)
NEWS2 Day 10	3	0.52	0.79	0.62	0.72	0.709 (0.62–0.80)

^1^ PPV = positive predictive value, ^2^ NPV = negative predictive value, ^3^ AUC = area under the curve, ^4^ CI = confidence index.

## Data Availability

The data presented in this study are available upon request from the corresponding author. The data are not publicly available due to containing information that could compromise the privacy of research participants.
